# Tai Chi for Stroke Rehabilitation: A Systematic Review and Meta-Analysis of Randomized Controlled Trials

**DOI:** 10.3389/fphys.2018.00983

**Published:** 2018-07-25

**Authors:** Diyang Lyu, Xuanxin Lyu, Yong Zhang, Yi Ren, Fan Yang, Li Zhou, Yihuai Zou, Zongheng Li

**Affiliations:** ^1^Neurological Rehabilitation Center, Beijing Rehabilitation Hospital, The Affiliated Hospital of Capital Medical University, Beijing, China; ^2^Department of Rehabilitation, Dongzhimen Hospital, The First Affiliated Hospital of Beijing University of Chinese Medicine, Beijing, China; ^3^Department of Neurology and Stroke Center, Dongzhimen Hospital, The First Affiliated Hospital of Beijing University of Chinese Medicine, Beijing, China

**Keywords:** Tai Chi, stroke, rehabilitation, systematic review, meta-analysis

## Abstract

**Background:** Stroke is a major cause of poor health and has numerous complications. Tai Chi (TC) may have positive effects on the rehabilitation of stroke survivors, but recent clinical findings have not been included in previously published reviews.

**Objectives:** We conducted this systematic review and meta-analysis to determine the effectiveness of all types of TC vs. conventional rehabilitation therapy for all aspects of stroke survivors' rehabilitation that have been studied.

**Method:** We searched seven electronic literature databases (three in English, four in Chinese) and one clinical registry platform using established strategies to identify randomized controlled trials performed up to October 2017. Screening, quality assessment, and data collection were performed by two researchers separately, using the same standard. The results were analyzed using RevMan 5.3.0. The quality of evidence was evaluated with GRADEpro.

**Results:** A total of 21 studies with 1,293 stroke survivors met inclusion criteria; 14 were included in the quantitative synthesis to evaluate four aspects and five outcomes. Nine studies indicated that TC was able to improve independent activities of daily living (ADL), especially TC vs. conventional rehabilitation therapy [mean difference (MD) [95% confidence interval (CI)] = 9.92 [6.82, 13.02], *P* < 0.00001]. Five studies reported significant effects of TC plus conventional rehabilitation therapy in increasing scores on the Fugl–Meyer Assessment for the upper limb [MD (95%CI) = 8.27 [4.69, 11.84], *P* < 0.0001], lower limb [MD (95%CI) = 2.75 [0.95, 4.56], *P* = 0.003], and overall [MD (95%CI) = 4.49 [1.92, 7.06], *P* = 0.0006]. The Berg Balance Scale revealed significant improvements according to pooled estimates for TC vs. conventional rehabilitation therapy [MD (95%CI) = 5.23 [3.42, 7.05], *P* < 0.00001]. TC plus conventional rehabilitation therapy also improved walking ability as measured by the Holden scale [MD (95%CI) = 0.61 [0.38, 0.85], *P* < 0.00001] and up-and-go time [MD (95%CI) = 2.59 [1.76, 3.43], *P* < 0.00001].

**Conclusion:** TC has an overall beneficial effect on ADL, balance, limb motor function, and walking ability among stroke survivors, based on very low-quality evidence, and may also improve sleep quality, mood, mental health, and other motor function. Well-designed, higher-quality trials with longer-term follow-up periods are needed to develop better-quality evidence.

## 1. Introduction

Stroke is a major cause of years lived with disability (YLDs) (GBD 2016 Disease and Injury Incidence and Prevalence Collaborators, 2017) and leads to the second-largest number of disability-adjusted life years (DALYs) lost (GBD 2016 DALYs and HALE Collaborators, 2017) worldwide. Stroke has high incidence, prevalence, and mortality, and results in many kinds of sequelae for survivors. Stroke also has a high rate of disability and mortality and is a huge burden to the people affected. Stroke can not only seriously impair the quality of life of survivors, but also leads to a huge financial burden on health services (Feigin et al., 2014). Although the global mortality rates of stroke reported in a recent study indicated a decrease, the number of cases of new-onset stroke, post-stroke disability, and stroke-related death have been increasing from 1990 to 2010 (Feigin et al., 2014). This is expected to continue to deteriorate over the next 20 years as the population ages and lifestyles change (Giroud et al., 2014).

Post-stroke rehabilitation often lasts from months to years, especially the rehabilitation of motor functions. Survivors suffer from language dysfunction, motor dysfunction, or other functional impairment affecting independent living ability; they often require family care and have a long process of recovery (Langhorne et al., 2011). The current proven rehabilitation techniques, which are widely used, have one thing in common: the need for a rehabilitation therapist to provide one-on-one treatment (Langhorne et al., 2011). However, stroke survivors and their families, especially in developing countries (Feigin et al., 2014), are usually unable to afford the cost required for a therapist or rehabilitation devices.

Tai Chi (TC), the practice of which has spread around the world in recent decades, was created in China and has been passed on for thousands of years. Also known as Tai Ji or Tai Chi Chuan as a form of physical activity, it focuses on controlling the stability of movements and the speed of breath, and increasing peace of mind; it also improves strength, flexibility, co-ordination, and balance, combining mind, body, and soul together in one workout (Li et al., 2001; Jahnke et al., 2010; Yang et al., 2015). It has also been proven to be effective in improving activities of daily living (ADL) of the elderly, and decreasing the rate of falls (Bubela et al., 2017). TC has been applied in stroke rehabilitation for over 10 years worldwide (Hart et al., 2004). Some clinical studies and reviews have demonstrated the safety and efficacy of TC for stroke survivors with dysfunctions, indicating that it may be of value in stroke rehabilitation. In recent years, some studies have confirmed the role of TC in improving motor function (Taylorpiliae et al., 2014) and balance (Chen et al., 2015) of stroke survivors. Some ongoing studies are investigating the effects of TC for stroke rehabilitation (Zhang et al., 2014; Tao et al., 2015), but the previously published systematic reviews and meta-analyses lack results from the increasing number of new studies. Besides, the experimental groups in clinical trials have involved either TC alone or TC combined with conventional rehabilitation therapy, but none of the previous reviews have mentioned this or divided the trials into two subgroups to conduct separate analyses. Here, we address these defects, and also combine several widely accepted clinical indicators together to give a more comprehensive conclusion and promote clinical decision-making. In systematically reviewing published articles on the effect of TC in stroke rehabilitation, we summarize and analyze various aspects, including ADL, mood or mental health, balance, motor function, and flexibility. This systematic review and meta-analysis provides information that may be able to help clinicians make evidence-based decisions regarding the application of TC in stroke rehabilitation.

## 2. Method

This review was performed according to our previous protocol (Zhang et al., 2016), which was registered on the international prospective register of systematic reviews, PROSPERO (http://www.crd.york.ac.uk/PROSPERO), registration number: CRD42015026999. Our study was performed to comply with the Preferred Reporting Items for Systematic Reviews and Meta-Analyses (PRISMA) statement (Moher et al., 2009). The methodological issues were solved with the guidance of the Cochrane Handbook for Systematic Reviews of Interventions (Higgins and Green, 2011).

### 2.1. Inclusion and exclusion criteria

We identified studies using the criteria below as in our protocol (Zhang et al., 2016):

1. Participants: stroke survivors were diagnosed according to the World Health Organization (WHO) definition (Listed, 1989) or confirmed by imaging. We accepted both ischemic and hemorrhagic stroke survivors, regardless of age, course of the disease, sex, or race.
2. Intervention: all types of TC were included in our study. Moreover, a TC group could be with or without conventional rehabilitation therapy treatment as in the corresponding control group, and studies of two designs were analyzed separately.
3. Comparison: control groups involved only conventional rehabilitation therapy treatment. Stroke diets, acupuncture, lifestyle modifications for stroke, and other co-interventions were acceptable only if being used in both groups of a trial.
4. Outcome: all of the outcomes available were included in our qualitative synthesis. The meta-analysis was conducted only when no less than two studies with similar study design reported the same outcome indicator.
5. Study design: we included only randomized controlled trials (RCTs), and excluded both quasi-randomized trials and other types of studies. Trials involving stroke survivors and using TC as the technique of rehabilitation met our criteria.

### 2.2. Search strategy

A computerized literature search was performed to find published reports on the effect of TC for stroke rehabilitation. The following electronic databases were searched using the developed search strategy (Zhang et al., 2016) from inception to 5 October 2017: MEDLINE, EMBASE, the Cochrane Library, the Chinese National Knowledge Infrastructure (CNKI), the Chinese BioMedical Literature Database (CBM), the Chinese Science and Technology Periodical Database (VIP), and Wanfang. We also searched the WHO International Clinical Trials Registry Platform (ICTRP) and its registry network for additional studies. Under the guidance of the Cochrane Handbook, two researchers searched all databases with the established strategy and screened the hit literatures individually using Endnote; the third investigator resolved disagreements between the initial two researchers if necessary.

### 2.3. Data extraction

For each trial, we carefully collected information for all eligible publications, including first author, year of publication, missing data, number of stroke survivors, withdrawn or dropped stroke survivors with the reason, sex ratio, mean age, duration of stroke, specific interventions for each group, frequency and duration of treatment, and all outcome measures. For studies with several different arms, we extracted the necessary data. For those with more than one time point to observe and assess, we chose to use the data as assessed at the end of the clinical treatment. Furthermore, we tried to contact every author for probable additional data, especially unpublished data. For those who refused to provide or confirm information, or whom we were unable to contact, we also recorded this information faithfully to provide a reference for the next step of quality assessment.

### 2.4. Quality assessment of RCTs

One investigator called every Chinese author and e-mailed the others regarding incomplete information and to confirm the methodological quality. For the theses, the investigator called the tutors to request the phone number of the author. For those who refused to answer any exact question or that we failed to get in touch with, we assessed their studies with the original information only, but noted that the missing information would lead to a “high risk of bias” in the corresponding aspects, as well as “other bias.” If no answer was obtained from the author after no fewer than five phone calls or two e-mails, an “unclear risk of bias” would be recorded for the corresponding aspects, as well as other bias. Then, two investigators independently evaluated the included articles. The methodological quality of RCTs was assessed using the Cochrane risk of bias tool recommended by the Cochrane Handbook (Higgins and Green, 2011) and the CONSORT criteria (Bian et al., 2011). A third investigator resolved any disagreements through discussion.

### 2.5. Statistical analysis

We used the Review Manager software (Revman5.3) for the meta-analysis. As almost none of the studies applied exactly the same set of TC, the random-effects model was utilized as recommended by the Cochrane Handbook, as there could be heterogeneity among the original studies that might not be shown by the data. The I^2^ statistic and the chi-squared test were also employed to evaluate the heterogeneity revealed by data analysis, and sensitivity analysis was performed to check the stability of the results by excluding each study one by one. This meta-analysis only included continuous data, so we used the mean difference (MD) and 95% confidence interval (CI) for analysis. We identified subgroups among studies according to their lengths of treatment and the total duration of treatment per week or during the whole trial, to explore factors that might be related to the strength of the effect. For those comparisons with huge heterogeneity that could not be explained by a subgroup analysis, the MD and 95%CI are not presented in the results.

### 2.6. Grade evaluation of evidence

As the Cochrane Handbook recommends, we applied the Grading of Recommendations Assessment, Development and Evaluation (GRADE) approach to evaluate the strength of our main outcomes. We used the five considerations recommended by the GRADE working group (Atkins et al., 2004)—study limitations, consistency of effect, imprecision, indirectness, and publication bias—to evaluate the quality of the evidence. As we only included RCTs, decisions to downgrade the quality of studies were recorded using footnotes and comments to describe the reasons. Summaries of findings for the main comparisons were created for studies involving TC with or without conventional rehabilitation therapy in the TC group.

## 3. Results

### 3.1. Results of the search

**Figure 1:**
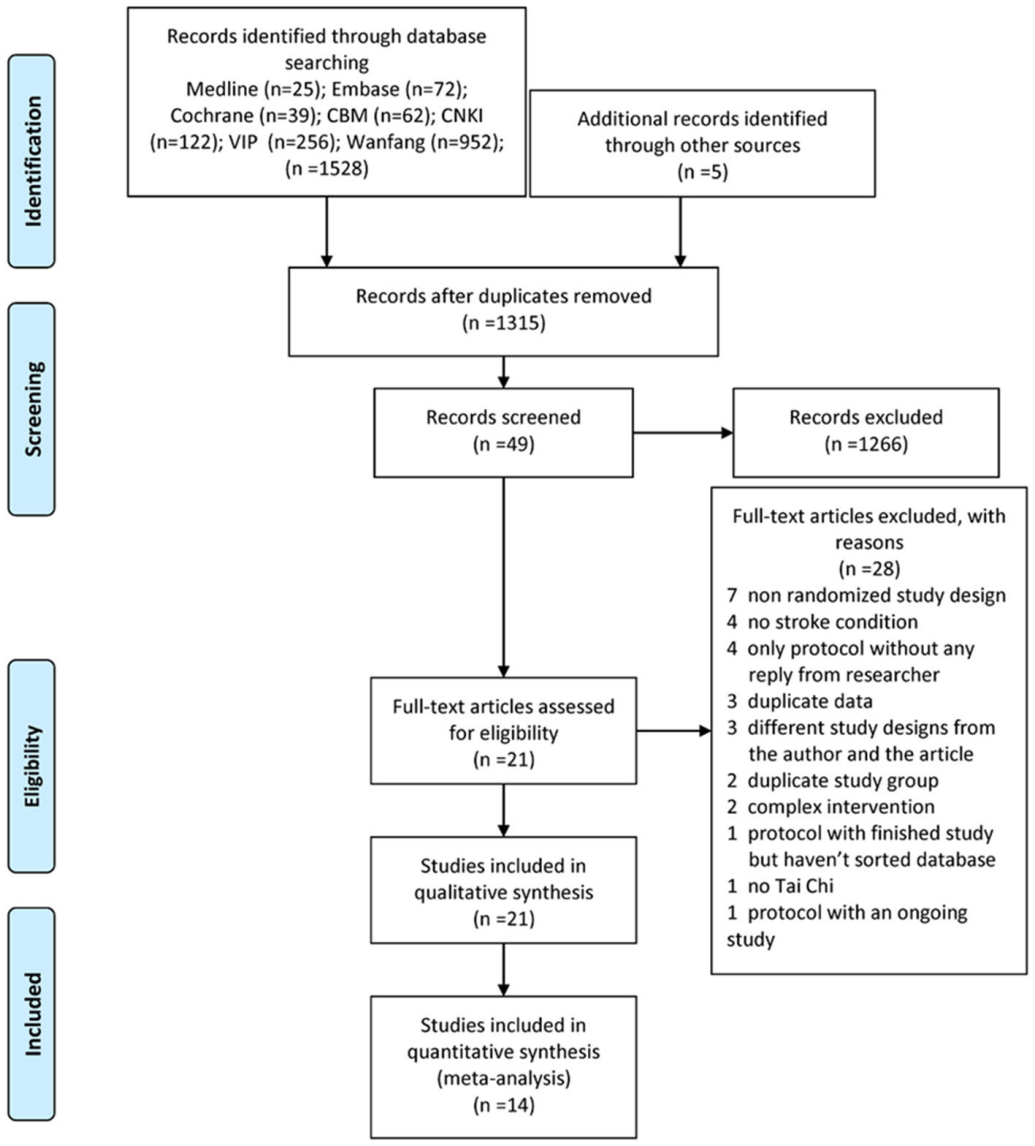
PRISMA 2009 flow diagram.

We systematically searched the electronic databases, and a total of 1,315 studies were identified after removing duplicates. We excluded 1,266 of these after examination of the titles and abstracts for at least one of following reasons: (i) no TC exercise; (ii) no stroke survivors; (iii) study design other than RCT. As well as reading the full article for the remaining 49 studies, we checked the reference lists for more reports not already included. Finally, we identified 21 eligible studies for inclusion in the systematic review and 14 for the meta-analysis after calling or e-mailing the authors to make sure the study met our inclusion criteria (Zhang et al., 2016) (Figure 1). Agreement between the review authors on exclusion was 100%.

### 3.2. Study characteristics

The 21 studies involved 1,293 stroke survivors (194 from six studies for TC alone, 404 from 16 studies for TC plus conventional rehabilitation therapy, and 585 from all 21 studies for the conventional rehabilitation therapy control), and 61 participants dropped out (36 from the TC group, 25 from the control group). Of the included studies, six were written in English and 15 were in Chinese; they were written by a total of 20 first authors and included one conference abstract. The studies were conducted across a range of continents and countries and were published from 2004 to 2017. Different types of TC were used as the intervention, and all of the control groups received CR. A total of 27 different scales or assessments were used in these studies. Notably, no adverse events were recorded. The basic characteristics of each RCT are presented in Table 1.

**Table 1:** Basic characteristics of the included studies.

**Study (Years)**	**Quantity (included plus dropped out), course, and intervention**	**Mean age and sex (male/female)**	**Ischemia/hemorrhage**	**Outcomes**	**Program (per W) and time points**
Hart et al., 2004	G1:9, NM, TC; G2:9, NM, NM.	G1:NM; G2:NM.	G1:NM; G2:NM.	BBS, TUGT	120 min. After 12 Ws.
Xie, 2008	G1:24, 3–20 days, CRT+TC-type rehabilitation; G2:24, 3–20 days, CRT.	G1:From 35 to 66 (NM); G2:From 38 to 62 (NM).	G1:NM (FO); G2:NM (FO).	BBS, BI	420–630 min. After 2 Ws.
Liu et al., 2009	G1:24, 17.65 ± 5.34 days, FR+simplified 24 types TC; G2:24, 17.65 ± 5.34 days, FR.	G1:52.13 ± 14.13 (14/10); G2:53.51 ± 12.63 (11/13).	G1:15/9 (FO); G2:16/8 (FO).	BBS	210 min. After 3 Ms.
Auyeung et al., 2009	G1:56+18 (NM, with older age), NM, 12 types TC; G2:52+10 (NM, with older age), NM, CRT.	G1:NM; G2:NM.	G1:NM; G2:NM.	TUGT, GBM	240 min. After 6 and 12 Ws treatment, 6-Ws follow-up.
Wang et al., 2010	G1:16, NM, Yang style TC; G2:13, NM, CRT.	G1:NM; G2:NM.	G1:NM; G2:NM.	P300, PSQI, General Health Questionnaire	G1: 50 min,G2: 80 min. After 12 Ws.
Li X. et al., 2011	G1:35, NM, CRT+TC imagination; G2:32, NM, CRT.	G1:56 ± 5.58 (NM); G2:54 ± 6.23 (NM).	G1:NM(FO); G2:NM(FO).	pinch strength, FMA-UE, BI	300 min. After 6 Ws.
Taylor-Piliae and Coull, 2012	G1:13+3 (2 other health issue, 1 death), NM, Simplified Yang style 24 types TC; G2:12, NM, FR+phone guide.	G1:72.8 ± 10.1 (7/6); G2:64.5 ± 10.9 (7/5).	G1:12/4 (14FO); G2:9/3 (10 FO).	SPPB, Number of steps in two min, SF-36, CES-D, PSQI	180 min. After 12 Ws.
Yang et al., 2013	G1:50, 44.7 ± 18.4 days, TC balance rehabilitation+TC balance 3 types; G2:50, 44.7 ± 18.4 days, CRT.	G1:54.3 ± 13.8 (35/15); G2:55.2 ± 14.6 (31/19).	G1:NM (FO); G2:NM(FO).	BBS, BI	270 min. After 1 M.
Zhou, 2013	G1:21+1 (lung cancer), with in 0.5 year, CRT+Modified TC; G2:19+3 (1 tuberculosis, 1 poor, 1 absence), with in 0.5 year, CRT.	G1:55.33 ± 9.47(19/2); G2:54.74 ± 7.02 (17/2).	G1+G2:40/0.	FMA, BBS, BI, Stroke Specific Quality of Life Scale	300 min. After 1 M.
Miu et al., 2014	G1:29, NM, CRT+Yang style 24 types; G2:28, NM, CRT.	G1:NM; G2:NM.	G1:NM (FO); G2:NM (FO).	GBM	420 min. After 8 Ws.
Taylorpiliae et al., 2014	G1:48+5(3 other disease, 1 moved away, 1 death), NM, Simplified Yang style 24 types TC; G2:45+3 (refuse to enter the control group), NM, FR+phone guide.	G1:71.5 ± 10.3 (34/19) (included the drop-outs); G2:68.2 ± 10.3 (23/25) (included the drop-outs).	G1:33/12 (8 unknown) for all (47FO); G2:30/14 (4 unknown) for all (42FO).	Fall-related data, SPPB, SF-36, PSQI, CES-D	180 min. After 12 Ws.
Kim et al., 2015	G1: 11,NM, CRT+10 types TC; G2: 11,NM, CRT.	G1: 53.45 ± 11.54 (7/4); G2: 55.18 ± 10.20 (6/5).	G1:NM; G2:NM.	GBM, 10m walking test, TUGT, SF-36, dynamic gait index, Functional reach test	G1: 120 min TC, G1&G2: 300 min CRT. After 6 Ws.
Zheng et al., 2015	G1:51+5, FO33, the others 18 included the dropped out, TC 10 types or non-stroke 24 types; G2:55+1, FO33, the others 18 included the dropped out, CRT.	G1:59 ± 13 (27/24); G2:60 ± 12(31/24).	G1+G2:106/0.	BI, HAMA, HAMD, NIHSS	210 min. After 3, 12 Ms.
Zhou, 2015	G1:34, 56.6 ± 14.4 days, Acupuncture+CRT+TC footwork; G2:32+2 (1 other disease, 1 left hospital), 56.6 ± 14.4 days, Acupuncture+CRT.	G1:62.6 ± 5.7(20/14); G2:63.3 ± 6.0 (22/10).	G1:28/6 (FO); G2:25/7 (FO).	FMA-LE, BBS, GBM, Holden, BI	G1: 200–300 min TC,G1&G2: 450 min CRT. After 6 Ws.
Yu et al., 2015	G1:40, NM, CRT+TC footwork; G2:40, NM, CRT.	G1:NM; G2:NM.	G1:NM; G2:NM.	Holden, BBS, FMA-LE	NM. After 4 Ws.
Fu and Zhang, 2016	G1: 30, within 3 Ms, CRT+simplified 24 types TC; G2: 30, within 3 Ms, CRT.	G1: 59.7 ± 7.6 (19/11); G2: 60.3 ± 8.4 (18/12).	G1: 17/13 (FO); G2: 20/10 (FO).	Trunk Impairment Scale, BBS, Holden, 10 m maximum walking speed	240 min. After 8 Ws.
Huang, 2016	G1: 8+1 (absence), 15.13 ± 7.30Ms, Yang style 6 types TC; G2: 8, 18.63 ± 31.47Ms, CRT.	G1: 65.00 ± 6.16 (6/2); G2: 63.63 ± 7.37 (7/1).	G1: 7/1; G2: 6/2.	SF-36, BBS, TUGT, Eyes-opened stand on one leg assessment, digital finger tapping test	120 min. After 24 Ws.
Wang X. et al., 2016	G1:14, 15.07 ± 8.51Ms, TC Yunshou; G2:16, 15.07 ± 8.51Ms, Balance CRT.	G1:60.71 ± 7.32 (9/5); G2:58.56 ± 8.52(14/2).	G1:11/3 (FO); G2:10/6 (FO).	BBS, GBM	300 min. After 12 Ws.
Wang, 2016	G1:25+2(NM), 5.50 ± 2.09Ms, CRT+TC Yunshou; G2:25+2(NM), 5.50 ± 2.09Ms, CRT.	G1:60.48 ± 8.29 (15/10); G2:60.92 ± 10.07 (16/9).	G1:NM (FO); G2:NM (FO).	BBS, TUGT, BI	150 min. After 4, 8 Ws treatment, 1 M follow-up
Yang et al., 2016	G1:30, within 3 Ms, TC balance rehabilitation+walking CRT; G2:30, within 3 Ms, Walking CRT.	G1:58 ± 11.27 (20/10); G2:60.07 ± 7.87 (21/9).	G1:NM (FO); G2:NM (FO).	BI, Holden, FMA-LE	420 min. After 1 M.
Zhao et al., 2017	G1:30, 40.58 ± 23.11 days, CRT+simplified 6 types TC; G2:30, 40.58 ± 23.11 days, CRT.	G1:53.85 ± 11.69 (20/10); G2:51.38 ± 14.83 (19/11).	G1:NM; G2:NM.	HAMD, BI, FMA	300 min. After 4 and 8 Ws.

*BBS, Berg Balance Scale; BI, Modified Barthel Index; CES-D, Center for Epidemiological Studies Depression scale; CRT, conventional rehabilitation therapy; FMA, Fugl-Meyer Assessment; FO, first onset; FR, Family rehabilitation; G1, Tai Chi group; G2, Control group; GBM, Gait biomechanical measurements; HAMA, Hamilton Anxiety Scale; HAMD, Hamilton Depression Scale; LE, Lower extremity; M, month; NIHSS, National Institute of Health stroke scale; NM, Not mentioned; PSQI, Pittsburgh Sleep Quality Index; RA, research article; SF-36, The 36-Item Short Form Health Survey; SPPB, Short Physical Performance Battery; TUGT, To up-and-go time; UC, Usual care; UE, Upper extremity; W, week*.

### 3.3. Risk of bias in included studies

**Figure 2:**
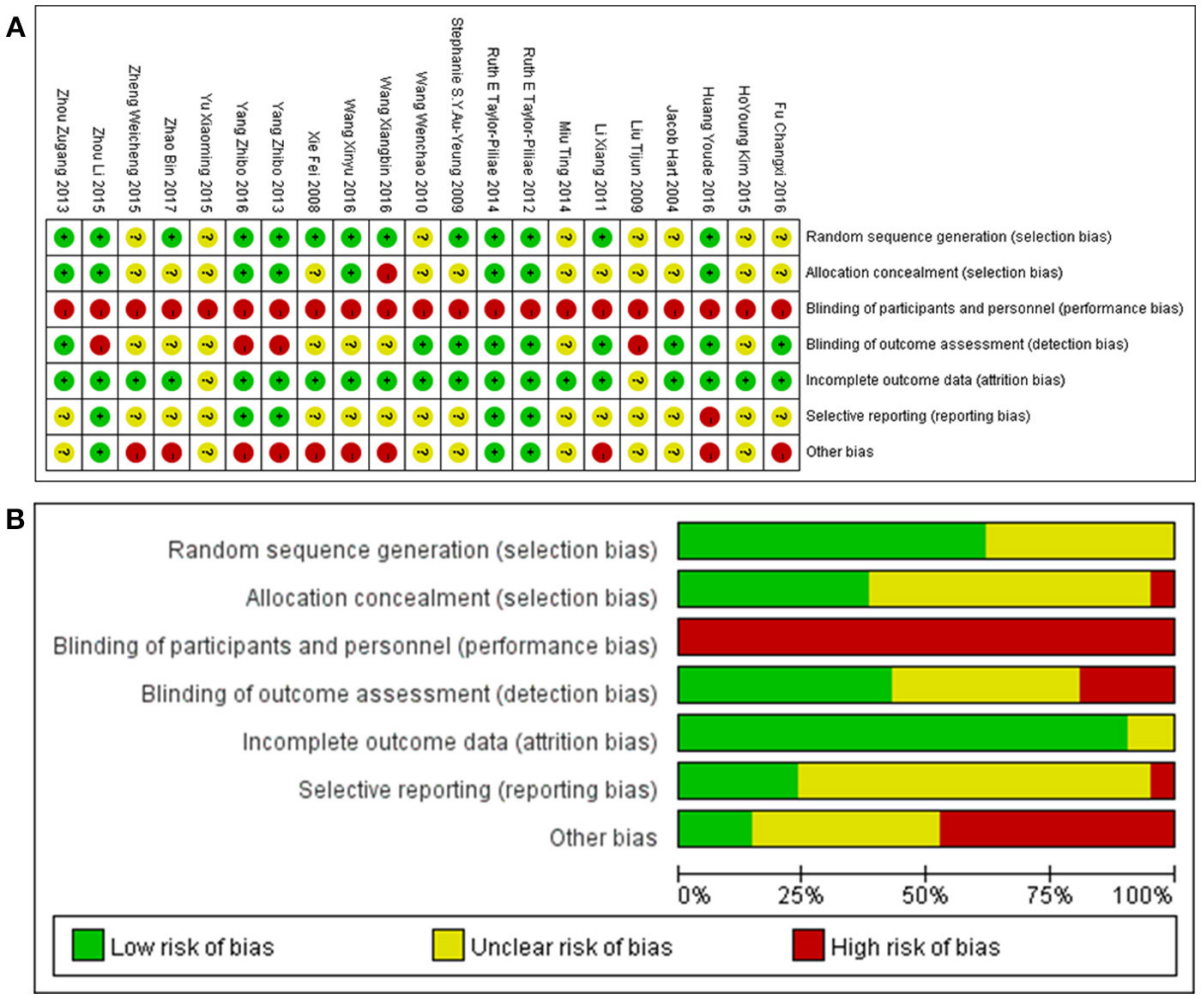
**(A)** Risk of bias summary: authors' judgments about each risk of bias item for each included study; **(B)** Risk of bias graph: authors' judgments about each risk of bias item presented as percentages across all included studies.

Risk of bias was assessed using the Cochrane risk of bias tool recommended by the Cochrane reviewers handbook and the CONSORT criteria (Bian et al., 2011). Eleven authors responded to telephone enquiries and one replied to our email enquiries. None of the authors contacted were able to provide additional data. Owing to the nature of TC, it was impossible to blind either stroke survivors or therapists. However, nine studies had blinding of outcome assessors. Most of the studies reported complete outcome data except the abstract. Following the phone calls and e-mails, we believed that five studies reported all outcomes, and the tutor of one thesis told us that they had some outcome data to report later; the others remained unclear. The full assessments of study quality are shown in Figure 2.

### 3.4. Qualitative description of the studies

On motor function, 21 studies used different evaluation methods to evaluate the effects of TC on different aspects of stroke recovery, including ADL, joint function, balance, walking ability, mood, and mental health.

#### 3.4.1. ADL

To explore whether TC had an effect on ADL, we used the modified Barthel Index (BI), the 36-Item Short Form Health Survey (SF-36), the Stroke Specific Quality of Life Scale (SS-QOL), the General Health Questionnaire (GHQ), the National Institute of Health Stroke Scale (NIHSS). Four publications out of 164 reported results from SF-36; although the authors recommended the use of TC, the results showed great inconsistency when considering the subcategories (Wang et al., 2010; Taylor-Piliae and Coull, 2012; Taylorpiliae et al., 2014; Huang, 2016). Significant advantages of TC were found using GHQ in one report of 29 stroke survivors (Wang et al., 2010) and NIHSS in another report of 106 stroke survivors compared with conventional rehabilitation therapy (Zheng et al., 2015); however, one publication reported no significant differences between the TC group (TC plus conventional rehabilitation therapy) and the control group that received conventional rehabilitation therapy, based on SS-QOL results for 40 stroke survivors (Zhou, 2015).

Nine publications measured BI results as an outcome of daily living quality; these consisted of seven studies of TC plus conventional rehabilitation therapy vs. conventional rehabilitation therapy with 391 stroke survivors (Xie, 2008; Li X. et al., 2011; Zhou, 2013, 2015; Wang, 2016; Yang et al., 2016; Zhao et al., 2017), and two studies of TC alone vs. conventional rehabilitation therapy with 166 stroke survivors (Yang et al., 2013; Zheng et al., 2015). Only one of these publications reported that there was no significant difference between TC plus conventional rehabilitation therapy and conventional rehabilitation therapy after treatment (Zhou, 2015); the others reported consistently that the TC group improved significantly in terms of daily living abilities compared with the conventional rehabilitation therapy group.

#### 3.4.2. Limb motor function

For limb movement and strength, the Fugl–Meyer Assessment (FMA), digital finger tapping test, and pinch strength test were employed. The results for pinch strength in one study of 67 stroke survivors showed significant differences for both two-finger and three-finger pinch (Li X. et al., 2011). The results of the digital finger tapping test in another study of 16 stroke survivors showed a significant difference for the TC groups intact forefingers after treatment; the affected forefingers improved as well, but the difference was not significant (*p* > 0.05) (Huang, 2016). Six studies in total used the FMA to determine the efficacy of TC treatment, including three studies of 246 stroke survivors that only assessed the lower limb (Yang et al., 2013; Zhou, 2013; Yu et al., 2015), one study of 67 stroke survivors that only assessed the upper limb (Li X. et al., 2011), and two studies of 100 survivors including both (Zhou, 2015; Zhao et al., 2017). The study that only assessed the score for the upper limb used the imagery techniques of TC Yunshou as the treatment, and paid no attention to the stroke survivors lower limbs (Li X. et al., 2011). The three publications that only reported significant differences for the lower limb consisted of one abstract (Yu et al., 2015), one report whose author told us there were no significant differences for the upper limbs in their study (Yang et al., 2016), and one report whose author we failed to contact (Zhou, 2015).

#### 3.4.3. Balance

To examine the effect on improving balance, researchers used seven different scales, namely the Berg Balance Scale (BBS), the functional reach test (FRT), the dynamic gait index (DGI), the eyes-open stand-on-one-leg assessment, the bodys center of pressure as determined by gait biomechanical measurements, fall-related data, and the Trunk Impairment Scale (TIS). In addition, biomechanical measurements were reported for balance in two studies. One study of 22 stroke survivors assessed using DGI, FRT, sway length, and velocity with eyes closed or open showed that TC might have an effect on dynamic and static balance; another study of 93 stroke survivors described the similar results by recording fall-related data. One study of 60 stroke survivors showed significant differences in TIS scores for dynamic and static balance and co-ordination when TC was used as an additional treatment (Kim et al., 2015). However, one study of 17 stroke survivors reported no significant statistical differences between groups, although an improvement was found within the TC group after treatment (Huang, 2016).

Up to 12 studies of 481 participants, including five who dropped out, with or without conventional rehabilitation therapy for the TC group, used BBS to measure both dynamic and static balance. Two studies of 70 stroke survivors showed improvement after treatment, but found no significant differences between groups; one study of 18 stroke survivors reported that TC had no effect on improving survivors balance, although conventional rehabilitation therapy did have an effect (Hart et al., 2004); and the other eight studies of 333 stroke survivors described significant differences both within and between groups (Xie, 2008; Liu et al., 2009; Zhou, 2013; Yu et al., 2015; Fu and Zhang, 2016; Huang, 2016; Wang, 2016; Yang et al., 2016).

#### 3.4.4. Walking ability

To explore the effect on walking ability, five different methods were used in 12 studies (Hart et al., 2004; Auyeung et al., 2009; Yang et al., 2013; Zhou, 2013; Miu et al., 2014; Taylorpiliae et al., 2014; Kim et al., 2015; Yu et al., 2015; Fu and Zhang, 2016; Huang, 2016; Wang, 2016; Wang X. et al., 2016), including the 10-m walking test, 10-m maximum walking speed test, up-and-go time (TUGT), the Holden scale, and gait biomechanical measurements. The 10-m walking test, 10-m walking speed test, and TUGT were used to measure walking speeds over 10 or 6 m. Two studies of 82 stroke survivors showed a larger improvement in the TC groups than the control groups with respect to 10-m walking speed (Kim et al., 2015; Fu and Zhang, 2016). Five studies of 219 stroke survivors reported varied results. One study of 18 stroke survivors from Israel in 2004 showed that the improvement in TUGT in the treatment group receiving only TC as rehabilitation was not significant, while in the control group that received group exercise as conventional rehabilitation therapy, the improvement in walking speed was significant (Hart et al., 2004). In one study of 108 stroke survivors conducted in Hong Kong (Auyeung et al., 2009), no significant differences were found in the within-group or between-group analyses. Three studies conducted in China demonstrated significant advantages of TC plus conventional rehabilitation therapy, based on improvements in TUGT scores of a total of 93 stroke survivors (Kim et al., 2015; Huang, 2016; Wang, 2016), although one of these studies reported no differences between groups after treatment (Huang, 2016). The Holden scale was used in four studies of 266 stroke survivors (Zhou, 2013; Yu et al., 2015; Fu and Zhang, 2016; Yang et al., 2016); the within-group and between-group analyses both showed significant improvements. Two studies of 123 stroke survivors using TC plus conventional rehabilitation therapy for the TC group (Zhou, 2013; Miu et al., 2014) and one study of 30 stroke survivors using only TC vs. conventional rehabilitation therapy (Wang X. et al., 2016) measured gait biomechanical outcomes. Only one of these reported significant differences on measures such as step length and walking speed after treatment, but did not report the data at baseline (Miu et al., 2014). One thesis on 66 stroke survivors reported significant improvements associated with TC in both within-group and between-group analyses (Zhou, 2013), and one study of 30 stroke survivors reported an improvement in walking ability after treatment in both groups, according to various measurements, but no significant differences were found between groups (Wang X. et al., 2016).

#### 3.4.5. General motor function

One additional outcome for motor functions was the Short Physical Performance Battery (SPPB), which measures walking function and static and dynamic balance using several tests. Two studies by the same researcher in America reported that stroke survivors in the TC group scored significantly better on balance, endurance, and quality of life after 12 weeks of treatment, compared with the control group who received family rehabilitation plus phone guidance. These changes were initially observed in the pilot study with only 25 stroke survivors (Taylor-Piliae and Coull, 2012), while in the later study involving 93 stroke survivors (including eight who dropped out), all scores increased significantly in both groups, with no differences found when intention-to-treat analyses were used (Taylorpiliae et al., 2014). The pilot study also measured the number of steps in 2 min as an outcome of endurance, showing that TC might be preferable to family rehabilitation plus phone guidance for this aspect of recovery.

#### 3.4.6. Mood and mental health

The evidence on mental and mood recovery was obtained using the Hamilton Anxiety Scale (HAMA), the Hamilton Depression Scale (HAMD), the Center for Epidemiological Studies Depression Scale (CES-D), P300, and the Pittsburgh Sleep Quality Index (PSQI). Three studies of 147 survivors used PSQI to evaluate the promotion of sleep quality, two of which reported the efficacy of TC in promoting sleep quality (Wang et al., 2010; Taylor-Piliae and Coull, 2012), although the third study, of 93 survivors, found no differences between groups or across time (Taylorpiliae et al., 2014). Two studies used HAMD and two used CES-D to determine the efficacy of TC in depression after stroke, and enrolled 284 stroke survivors. Two studies of 85 survivors receiving TC plus conventional rehabilitation therapy in the TC group showed that TC might have effect on depression (Taylor-Piliae and Coull, 2012; Zhao et al., 2017); no significant differences were found in the other two studies of 199 survivors (Taylorpiliae et al., 2014; Zheng et al., 2015). However, there may have been an improvement in anxiety (Zheng et al., 2015). The results of one study using P300 also suggested that TC might have a positive effect on survivors cognitive function (Wang et al., 2010).

### 3.5. Meta-analysis results

Based on the previous protocol (Zhang et al., 2016) and the summary of all outcome indicators included in the 22 studies from the literature, we chose the following five outcome measures for the meta-analysis: BI, FMA, BBS, Holden, and TUGT.

#### 3.5.1. BI

**Figure 3:**
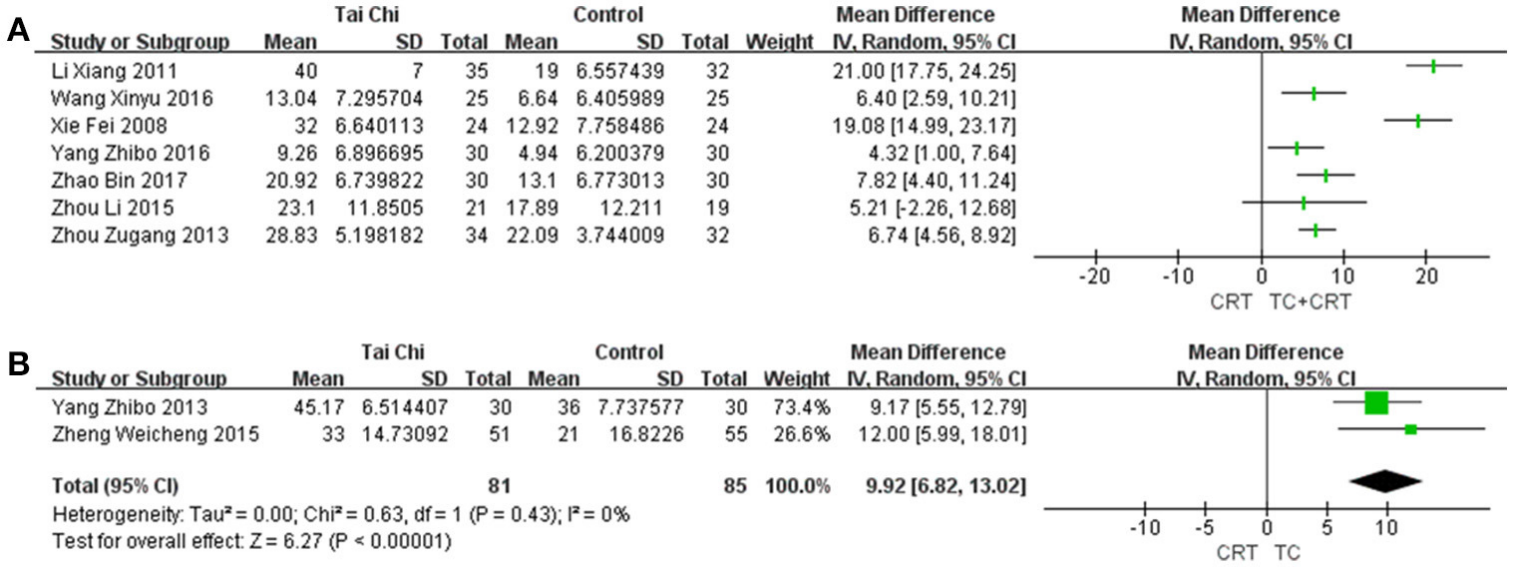
Forest plots of BI for **(A)** the studies comparing TC plus conventional rehabilitation therapy with conventional rehabilitation therapy alone, with 95%CI for each trial individually; and **(B)** the studies comparing TC with conventional rehabilitation therapy, with MD (95% CI) in a random-effects model. SD, standard deviation; IV, inverse variance.

BI was first proposed in 1965 (Mahoney and Barthel, 1965), and is widely used to evaluate the patients abilities in daily living. The scale contains 10 items, including feeding, moving from wheelchair to bed and return, and bathing self. The score measures whether the patient can complete the task independently or with limited help; if they are totally dependent on help, a score of 0 is given for all of the included activities. Data were available from nine trials, as shown in Figure 3, including two trials that studied TC vs. conventional rehabilitation therapy, and seven that looked at TC plus conventional rehabilitation therapy vs. conventional rehabilitation therapy. Significant improvements were found in the TC group vs. conventional rehabilitation therapy [MD (95%CI) = 9.92 [6.82, 13.02], *P* < 0.00001, *I*^2^ = 0%], according to two clinical trials. The comparisons of TC plus conventional rehabilitation therapy vs. conventional rehabilitation therapy showed huge heterogeneity [*I*^2^ = 93%], which and the subgroup analyses we performed were unable to explain. This may have been related to some substantial differences between the original studies in terms of their study designs. With sensitivity analysis, the result was stable, but the large heterogeneity still existed. For this reason, only the MDs (95%CI) of each individual study are presented in Figure 3A.

#### 3.5.2. FMA

**Figure 4:**
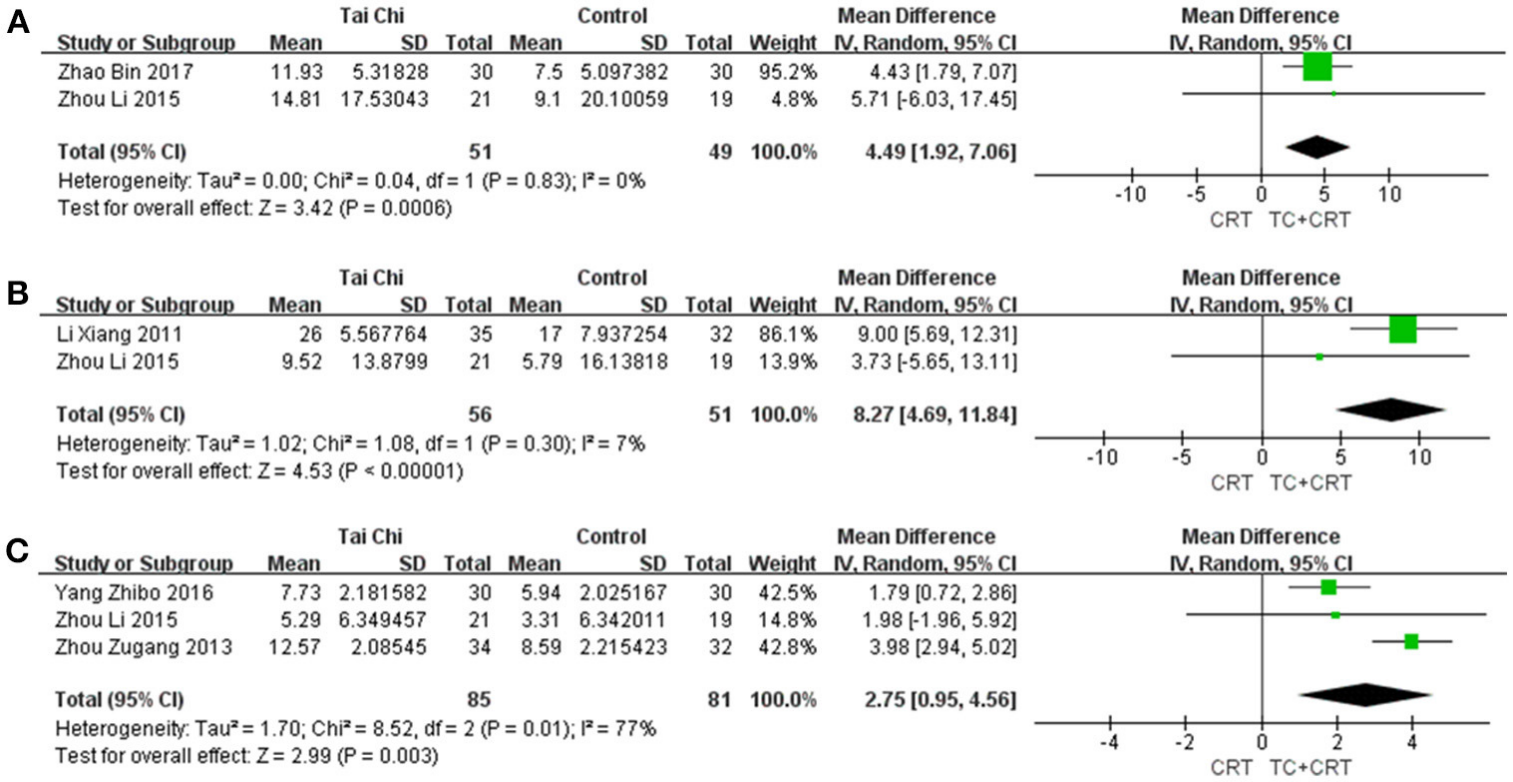
Forest plots showing MD (with 95% CI) for joint movement of the studies comparing TC plus conventional rehabilitation therapy with conventional rehabilitation therapy alone using FMA for **(A)** all four limbs; **(B)** the upper limb; **(C)** the lower limb.

FMA was first proposed in 1975 (Fuglmeyer et al., 1975), and is usually applied to evaluate motor function of the upper extremity (UE) and lower extremity (LE). The measure contains 50 items, with a total score of 100 points (UE 66, LE 34). The more serious the patients motor dysfunction, the lower the score. All the studies that used FMA for assessment compared TC plus conventional rehabilitation therapy with conventional rehabilitation therapy as a control. We merged the data separately for the upper limb, lower limb, and overall. As shown in Figure 4, three studies were merged to evaluate the motor function of LE [MD (95%CI) = 2.75 [0.95, 4.56], *P* = 0.003, *I*^2^ = 77%], two were merged for overall results [MD (95%CI) = 4.49 [1.92, 7.06], *P* = 0.0006, *I*^2^ = 0%], and two were merged for UE function [MD (95%CI) = 8.27 [4.69, 11.84], *P* < 0.00001, *I*^2^ = 7%]. For one trial, no significant differences were found (Yang et al., 2016), and the author declined to offer any additional data. Otherwise, each comparison showed a significant difference, indicating that TC plus conventional rehabilitation therapy might be more efficient than conventional rehabilitation therapy for survivors with motor dysfunction, whether for upper limbs, lower limbs, or overall. However, for the UE and overall comparisons, the results might be unreliable, because only two studies were included in each case.

#### 3.5.3. BBS

**Figure 5:**
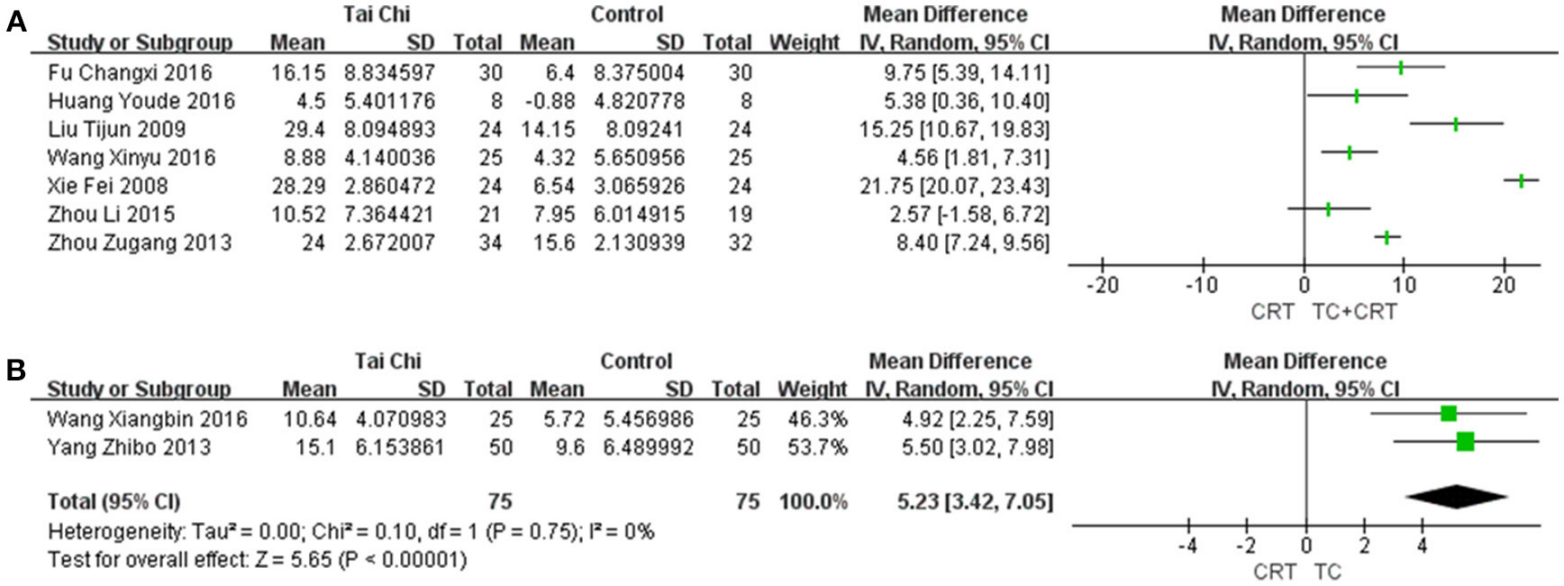
Forest plots for BBS of the studies **(A)** comparing TC plus conventional rehabilitation therapy with conventional rehabilitation therapy alone with 95%CI for each trial individually; and **(B)** comparing TC with conventional rehabilitation therapy with MD (with 95% CI) in a random-effects model.

Initially developed in 1987 (Berg, 1987) and validated in 1992 (Berg et al., 1992), BBS has become the most commonly used tool to assess the balance function of stroke survivors (Blum and Korner-Bitensky, 2008). This scale consists of 14 items, with four points for each item. The more help an individual needs to complete each task, the lower their score. Those with a total score of less than 40 points are more likely to fall; those scoring 21 to 40 require help to walk; those scoring less than 20 are in need of a wheelchair; and people with a score higher than 40 can be completely independent. As Figure 5 shows, in the recovery of balance, two trials compared TC with conventional rehabilitation therapy, implying a clear advantage for TC [MD (95%CI) = 5.23 [3.42, 7.05], *P* < 0.00001, *I*^2^ = 0%]. We also conducted a meta-analysis for the comparison between TC plus conventional rehabilitation therapy vs. conventional rehabilitation therapy alone; the pooled estimate showed huge heterogeneity [*I*^2^ = 97%], although the combined result indicated a positive effect for TC plus conventional rehabilitation therapy for stroke rehabilitation. We conducted subgroup analyses by frequency, total duration, duration per week, and total number of weeks of treatment. A sensitivity analysis was also performed, but did not lead to significantly less heterogeneity. Owing to the huge unexplained heterogeneity, only the MDs (95%CI) of each individual study are presented in Figure 5A.

#### 3.5.4. Holden and TUGT

**Figure 6:**
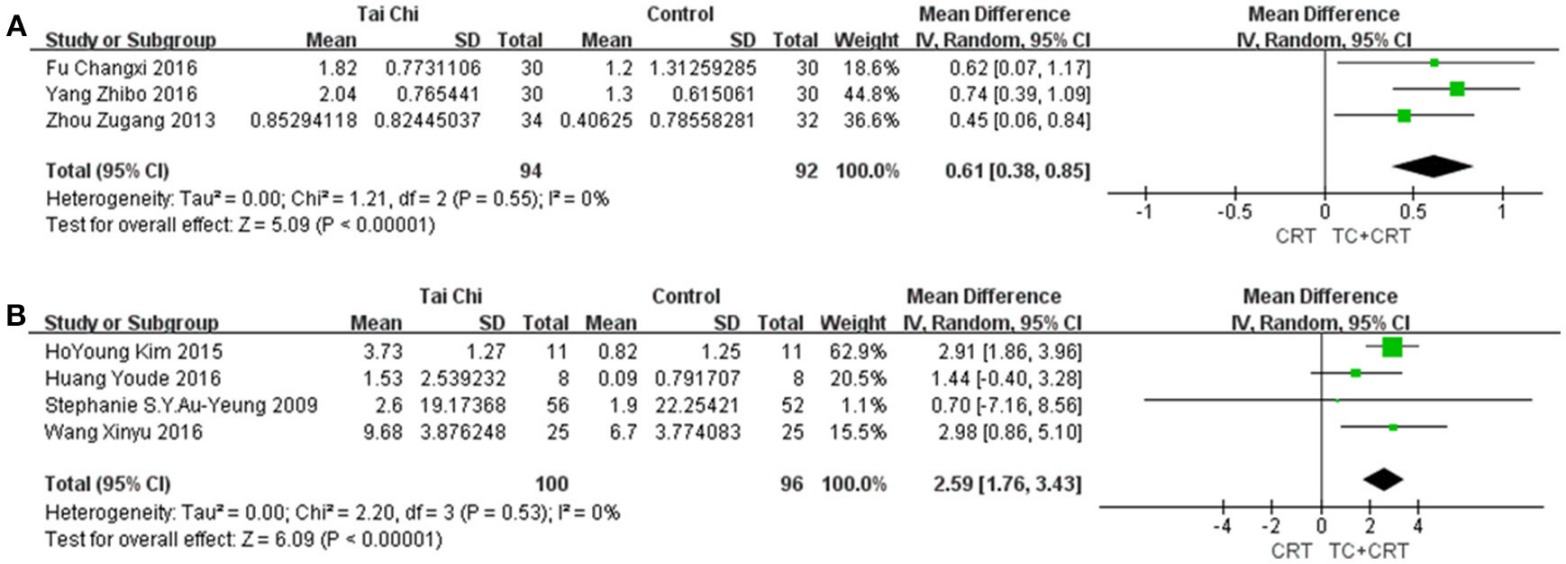
Forest plots showing MD (with 95% CI) for walking ability for the studies comparing TC plus conventional rehabilitation therapy with conventional rehabilitation therapy alone, using **(A)** the Holden scale; **(B)** TUGT.

To evaluate walking ability, two outcomes were used, as shown in Figure 6. First, we used a functional ambulation category scale named Holden, in which six levels are defined on the basis of a patients walking ability, from 0 to 5. Level 5 always represents normal walking ability (Holden et al., 1984). Second, we used TUGT, which was originally developed to measure the balance of elderly people. This instrument requires stroke survivors to stand up from a chair, walk 3 m, walk back, and sit down again, and records the time taken to do so (Mathias et al., 1986). Nowadays, this test is also widely used to measure stroke survivors walking ability. Three studies using Holden were included in the comparison, and four studies using TUGT. The pooled meta-analysis of the data indicated that both the Holden group [MD (95%CI) = 0.61 [0.38, 0.85], *P* < 0.00001, *I*^2^ = 0%] and TUGT group [MD (95%CI) = 2.59 [1.76, 3.43], *P* < 0.00001, *I*^2^ = 0%] showed marked effects of TC on survivors' rehabilitation.

### 3.6. Quality of the evidence

We graded the quality of the evidences as very low for all outcomes. According to the risk of bias summary (Figure 2A) and graph (Figure 2B), there was a strong risk of bias relating to study design and incomplete reporting. The nature of TC and rehabilitation made it impossible to apply a double-blind study design. Moreover, only nine studies mentioned blinding of assessors; seven of these contributed data to the meta-analysis, but the others were unclear. In addition, 13 studies described a method to generate a random allocation sequence, and eight reported an approach to achieve allocation concealment. From the results of the meta-analysis, we found that no single outcome had a combined total of more than 400 stroke survivors (Guyatt et al., 2011). To evaluate the quality of the original studies and obtain more data, we contacted all available first and corresponding authors: some refused to answer any questions on their studies; some answered our questions but did not offer any more data, although they claimed there were unpublished data, including negative results; and the others we failed to get in touch with. We did subgroup analyses based on the number of treatment weeks, the weekly treatment frequency, the weekly treatment duration, and the total treatment duration, but none of these was found to correlate with efficacy, and there were still large heterogeneities among the studies. Nevertheless, most studies showed that TC might have a potential advantage in stroke rehabilitation compared with conventional rehabilitation therapy, and the results of the studies on each outcome showed good agreement in the meta-analyses. In summary, the results should be interpreted with caution; see the GRADE evidence profile (Tables 2, 3).

**Table 2:** GRADE evidence profile of Tai Chi plus conventional rehabilitation therapy vs. conventional rehabilitation therapy.

**Quality assessment**							**No of patients**		**Effect**		**Quality**	**Importance**
**No of studies**	**Design**	**Risk of bias**	**Inconsistency**	**Indirectness**	**Imprecision**	**Other considerations**	**Tai Chi plus conventional rehabilitation therapy**	**Control**	**Relative (95% CI)**	**Absolute**		
**BBS (follow-up 2–24 weeks; measured with: the Berg Balance Scale; Better indicated by lower values)**												
7	Randomized trials	Serious^a^	No serious inconsistency	No serious indirectness	Serious^b^	Reporting bias^c^	166	162	–	Not pooled	⊕OOO VERY LOW	CRITICAL
**BI (follow-up 2–8 weeks; measured with: the modified Barthel Index; Better indicated by lower values)**												
6	Randomized trials	Serious^a^	No serious inconsistency	No serious indirectness	Serious^b^	Reporting bias^c^	165	160	–	Not pooled	⊕OOO VERY LOW	CRITICAL
**FMA (follow-up 4-8 weeks; measured with: the Fugl-Meyer Assessment; Better indicated by lower values)**												
2	Randomized trials	Serious^a^	No serious inconsistency	No serious indirectness	Serious^b^	Reporting bias^c^	51	49	–	MD 4.49 higher (1.92 to 7.06 higher)	⊕OOO VERY LOW	CRITICAL
**FMA-UE (follow-up 4-6 weeks; measured with: the Fugl-Meyer Assessment of upper extremity; Better indicated by lower values)**												
2	Randomized trials	Serious^a^	No serious inconsistency	No serious indirectness	Serious^b^	Reporting bias^c^	56	51	–	MD 8.27 higher (4.69 to 11.84 higher)	⊕OOO VERY LOW	CRITICAL
**FMA-LE (follow-up 4-6 weeks; measured with: the Fugl-Meyer Assessment of lower extremity; Better indicated by lower values)**												
3	Randomized trials	Serious^a^	No serious inconsistency	No serious indirectness	Serious^b^	Reporting bias^c^	85	81	–	MD 2.75 higher (0.95 to 4.56 higher)	⊕OOO VERY LOW	CRITICAL
**Holden (follow-up 4-8 weeks; measured with: Holden walking function score; Better indicated by lower values)**												
3	Randomized trials	Serious^a^	No serious inconsistency	No serious indirectness	Serious^b^	Reporting bias^c^	94	92	–	MD 0.61 higher (0.38 to 0.85 higher)	⊕OOO VERY LOW	CRITICAL
**TUGT (follow-up 6-24 weeks; measured with: To Up-and Go time; Better indicated by lower values)**												
4	Randomized trials	Serious^a^	No serious inconsistency	No serious indirectness	Serious^b^	Reporting bias^c^	100	96	–	MD 2.59 higher (1.76 to 3.43 higher)	⊕OOO VERY LOW	CRITICAL

a *Downgraded one level because of risk of bias: none of the studies applied double-blind design, most of whom also ignored to blind assessor, some didn't report the method used to generate the random allocation sequence or to achieve allocation concealment*.

b *Downgraded one level because of imprecision: the total number of the participants is < 400*.

c *Downgraded one level due to a possible publication bias: none had been enrolled on a platform for clinical trials, and no researcher agreed to share any more results except of those in their literatures, though some of them told us exactly the unpublished data existed*.

**Table 3:** GRADE evidence profile of Tai Chi vs. conventional rehabilitation therapy.

**Quality assessment**							**No of patients**		**Effect**		**Quality**	**Importance**
**No of studies**	**Design**	**Risk of bias**	**Inconsistency**	**Indirectness**	**Imprecision**	**Other considerations**	**Tai Chi without conventional rehabilitation therapy**	**Control**	**Relative (95% CI)**	**Absolute**		
**BBS (follow-up 4–12 weeks; measured with: the Berg Balance Scale; Better indicated by lower values)**												
2	Randomized trials	Serious^a^	No serious inconsistency	No serious indirectness	Serious^b^	Reporting bias^c^	75	75	–	MD 5.23 higher (3.42 to 7.05 higher)	⊕OOO VERY LOW	CRITICAL
**BI (follow-up 4–52 weeks; measured with: the modified Barthel Index; Better indicated by lower values)**												
2	Randomized trials	Serious^a^	No serious inconsistency	No serious indirectness	Serious^b^	Reporting bias^c^	81	85	–	MD 9.92 higher (6.82 to 13.02 higher)	⊕OOO VERY LOW	CRITICAL

a *Downgraded one level because of risk of bias: none of the studies applied double-blind design, most of whom also ignored to blind assessor, some didn't report the method used to generate the random allocation sequence or to achieve allocation concealment*.

b *Downgraded one level because of imprecision: the total number of the participants is < 400*.

c *Downgraded one level due to a possible publication bias: none had been enrolled on a platform for clinical trials, and no researcher agreed to share any more results except of those in their literatures, though some of them told us exactly the unpublished data existed*.

## 4. Discussion

### 4.1. Main findings

Our meta-analysis found that whether or not it was used in conjunction with conventional rehabilitation therapy, there was evidence that TC could improve ADL, limb motor function, balance, and walking ability in stroke survivors. Furthermore, several studies reported improvements in different kinds of mental disorders with the use of TC. These results showed that stroke survivors receiving TC as rehabilitation in the experimental group recovered better compared with those receiving only conventional rehabilitation therapy in the control group; however, we did not find that any specific style or practice program of TC was related to the efficacy through subgroup analyses. We failed to reach a conclusion about which type of TC or approach to managing the treatment was more effective based on the present meta-analysis. TC with and without conventional rehabilitation therapy both showed an advantage in comparison with conventional rehabilitation therapy.

### 4.2. Interpretation of the results

Past clinical studies of TC have found that external factors affect stroke rehabilitation. Balance, limb movement, and gait are all related to muscle strength, especially of the lower limb. One of the latest studies showed an improvement of neuromuscular reaction using electromyography to analyze the changes in muscle function of the lower limbs of elderly women after practicing TC for a year (Sun et al., 2016); another study examining 3D kinetics proved that TC could lead to similar mechanical behavior of biological tissues as in healthy people and could strengthen the lower limbs, helping to improve walking ability and balance, and prevent falls in the elderly (Li and Law, 2018). For those suffering from lower limb paralysis, wheelchair TC also revealed a remarkable improvement in upper limb movement, such as the range of motion at the shoulder (Wang Y.T. et al., 2016). There are sufficient reasons for us to believe that TC improves the motor function of limbs, with indirect effects on balance, walking ability, and limb movement. Furthermore, ADL depends on muscle strength and joint mobility, which affect whether patients can go up or downstairs, dress, eat, or wash independently, and is also related to their cognitive ability to understand and carry out the tasks; these factors have also been used to confirm the efficacy of TC in recent years (Siu and Lee, 2018).

To investigate the internal and central mechanism, several studies used functional MRI (fMRI) and electroencephalography (EEG). Chen Lidian and his team discovered that the TC group had enhanced functional connectivities and amplitude of low-frequency fluctuations in several different brain areas and networks, including the bilateral dorsolateral prefrontal cortex and hippocampus, indicating that TC should be effective in preventing memory and cognitive decline (Tao et al., 2016, 2017). Another two studies suggested a significant improvement in older people's attention, which might also help to reduce falls, and improve balance and walking ability, by detecting meaningful functional and structural changes in the default mode network (Wei et al., 2013, 2014). The EEG study reported a larger P3b event-related potential switch trial amplitude, suggesting a benefit to executive function and indicating that TC might have a central effect in promoting recovery of the peripheral nervous system (Hawkes et al., 2014).

TC also had an effect on patient's immune system, via a potential mechanism of DNA repair and promotion of renewal and regeneration of lymphocytes in older adults; this might help to prevent replicative senescence and promote recovery from stroke (Goon et al., 2008).

In summary, TC is a complicated system. It might have peripheral and central mechanisms. TC can improve advanced cortical function, muscle strength, joint range of motion, and neuromuscular reaction through effects on the central nervous system and humoral system, promoting rehabilitation in stroke survivors. However, understanding of the mechanism is still deficient.

### 4.3. Reliability of the results

Our study reports positive effects of TC on ADL, balance, limb motor function, and walking ability, as demonstrated by meta-analysis. The results of previous studies are also summarized qualitatively to show the efficacy with respect to joint movement and strength, cognitive function, and mental health and mood recovery, as well as to corroborate the results of the meta-analyses.

#### 4.3.1. ADL

A previous systematic review (Li et al., 2014) summarized the effects of TC on health-related quality of life in patients with various chronic medical conditions. The review included a total of 1,200 patients in 21 RCTs (up to December 2013) conducted in five countries, which focused on diseases of the cardio-cerebrovascular and respiratory systems, musculoskeletal system, and endocrine and immune systems, as well as cancer. There were no limitations on the style of TC. For the TC groups, the original studies described TC plus usual care, several kinds of physical therapies, health education or TC alone for TC groups. TC was removed to form the corresponding control groups. Most of the RCTs used the SF-36 to evaluate quality of life; 18 of 21 studies found dramatic improvements in the TC groups, but none were able to show the superiority of TC over other types of exercise. A focused review summarizing clinical trial evidence for the effectiveness of TC for stroke rehabilitation in 2012 (Ding, 2012) included five RCTs in total, four of which reported an improvement in ADL. We included three of these studies in the present review; the other study was excluded as its author had been unable to describe their protocol or answer our questions, and even tried to lie during the phone call.

Our results were basically consistent with those of the previous reviews, and we additionally conducted a meta-analysis to evaluate ADL with BI. We report a preliminary finding that there is evidence of very low grade that TC might be more effective than conventional rehabilitation therapy in improving ADL, but we could not reach a conclusion as to whether TC was superior to yoga or other types of exercises in stroke rehabilitation.

#### 4.3.2. Limb motor function

Limb motor function is usually regarded as a combination of strength and range of motion. A previous meta-analysis (Liu et al., 2011) reported significant effects on lower limb myodynamia in elderly people after TC exercise compared with control groups that received no special treatment. The study involved two RCTs and two non-RCTs with 163 participants, and demonstrated that TC could enhance ankle and knee muscle by measuring maximum voluntary isometric contraction with an isokinetic dynamometer. The enhancement of ankle and knee muscle could improve both balance and walking ability. However, there were not enough reviews of TC for joint function. Our results for lower limb function were consistent with previous results, after including more newly published studies than before, and analyzing both the upper limb and all limbs in meta-analyses with FMA.

#### 4.3.3. Balance

Balance and fall prevention have been researched by many experts. The latest systematic review and meta-analysis (Chen et al., 2015) of traditional Chinese exercise on balance after stroke included nine studies, six of which used TC, while two used Baduanjin and one used Yijinjing. Six studies were included in the meta-analysis, and the primary outcome was BBS. In this study, the authors did not perform subgroup analysis based on whether the experimental groups used traditional Chinese exercise combined with the control group program. The review provided evidence of very low grade that traditional Chinese exercise had beneficial effects on balance ability in the short term. Our study identified a total of eight original studies and analyzed these individually according to the program of the TC groups; although we also found that the evidence was of very low grade, our conclusion might be more reliable and stable.

Another recent meta-analysis (Lomas-Vega et al., 2017) on TC provided high-quality evidence of a medium protective effect on fall incidence over the short term for older and at-risk adults. More studies are required regarding injurious falls and time to first fall. Our results were essentially the same as those of previous reviews, indicating that further high-quality studies are required.

#### 4.3.4. Walking ability

The application of the Holden scale was rare. A systematic review and meta-analysis (Huang and Liu, 2015) reported TC's effects on improving TUGT, with selected older adults as the population, and compared TC with other interventions. A total of seven RCTs were included; the pooled estimate indicated that TC significantly shortened the completion time of TUGT, although the improvement was weaker than in our study, possibly owing to a lower baseline level and probable recovery with the progress of the disease in stroke survivors.

#### 4.3.5. Other aspects

Post-stroke depression and anxiety are common symptoms. Previous research (Wang et al., 2014) has demonstrated the effects of TC in various populations on a range of psychological well-being measures, by analyzing a total of 37 RCTs and five quasi-experimental trials in chronic diseases including stroke and cancer.

For cognitive function, a meta-analysis (Northey et al., 2017) with 36 RCTs showed that physical exercise had positive effects, and also proved that a medium duration (45–60 min) might be better than a short or long duration of exercise, although only four studies involved TC for adults older than 50. Our study described P300 as a central biological indicator; this is a potential future research direction for cognition impairment.

Another research group (Du et al., 2015) performed a meta-analysis with five RCTs, providing weak evidence of a decrease in the global PSQI score in older people practicing TC vs. control groups receiving no treatment. This result was different from our summary of included studies, possibly owing to post-stroke central injury or too much rest time during treatment.

#### 4.3.6. Simple summary

As the study of TC in clinical trials worldwide is a relatively recent development, most previous reviews, like ours, described most of the evidence as “weak” or of “very low” quality. The results generally suggested that TC may have beneficial effects in stroke survivors and the elderly, but more high-quality original clinical studies are still needed. As the requirements for the quality of original research become more demanding, and the Data Sharing Statements for Clinical Trials continue to be being promoted (Taichman et al., 2017), it will be more feasible to evaluate the authenticity, scientificity, objectivity, and reliability of the original studies in the future, to determine the possible causes of the differences between studies, and to increase the grade of the evidence available.

### 4.4. Implications for tai chi practice

This systematic review and meta-analysis demonstrates the efficacy of TC on stroke rehabilitation. Depending on our results, stroke survivors receiving TC comprehensively exhibit significant amelioration in post-stroke neurological function deficits. Of course, therapists and doctors should pay attention to adverse events, although none was reported in any of these 21 publications, and a previous study also confirmed that TC is unlikely to cause serious adverse events (Wayne et al., 2014). However, TC exercise leads to stroke survivors engaging in independent activity without passive support, which might be associated with a certain risk of falls or over-fatigue.

### 4.5. Implications for further clinical researches

There are a few points that clinical researchers should consider in the future (Li J. Y. et al., 2011). First, the methodological quality of RCTs needs to be improved; more studies with rigorous design and normative description are needed in this field, which requires researchers to study the principles of evidence-based medicine more seriously. In addition, studies should take into account characteristics of patients, such as whether they have cerebral hemorrhage or cerebral ischemia, different duration of stroke from the first onset, and different age ranges, to refine the study of TC further. We expect to see research on the efficacy of different TC styles, the efficacy of TC in combination with conventional rehabilitation therapy or alone, and even the study of modified TC for stroke rehabilitation. A previous review (Wu et al., 2016) aimed at improving balance in older adults pointed out that there are often missing details on exercise prescription and instructional methods; this might be a flaw in most of the research in this area. To reduce selective reporting and develop the efficiency of all research, pre-registration on an international platform might be advisable for a clinical trial. To make the outcomes and results of further clinical research more reliable and objective, it will be necessary to apply advanced assessment instruments such as the gait scanner to introduce biomechanical indicators into studies, just as the previous three studies included in this review did (Zhou, 2013; Miu et al., 2014; Wang X. et al., 2016). However, the differences between instruments and the selection of specific evaluation indicators still need further research.

### 4.6. Implications for further evidence-based studies

One study protocol (Anne Harkin et al., 2016) and five ongoing studies met the inclusion criteria of a this review [NCT03252236, NCT01848080 (Tousignant et al., 2014), ChiCTR-TRC-13003641 (Tao et al., 2015), ChiCTR-TRC-13003661 (Zhang et al., 2014), ChiCTR-TRC-13003601]. Of these, three were conducted on the Chinese mainland, one in Hong Kong, and one in Canada. This proportion is similar to the proportion of all the studies included in the review. The authors of another abstract included in our review might report their detailed data in the future, while another had promised to write articles later (Yu et al., 2015).

### 4.7. Limitations

There were several limitations in this study. First, we only searched and included the literature in Chinese and English, while there might be some relevant studies in Japanese, Korean, and other languages. Second, we failed to get in touch with all authors to request more data and evaluate the methodological quality, and so about one-third of the risk of bias graph remains unclear. Finally, owing to the insufficient quantity of studies included, we failed to elucidate the relationship between the efficacy and any of the factors, such as the type of TC and the frequency of treatment.

## 5. Conclusion

In summary, this review suggests that TC may have effects on improving ADL, balance, limb motor function, and walking ability in stroke survivors, with evidence of very low quality, and may also improve their sleep quality, mood, mental health, and other motor functions. In addition, no adverse events were reported among the included studies. More longer-term, well-designed and high-quality trials are needed to continue developing the quality of evidence in this worthwhile field.

## Author contributions

YoZ, ZL, and XL designed the systematic review. YR and DL did the literature search, reviewed all the publications. FY and XL extracted the information and data from the included studies. DL and LZ did the data analysis and produced the figures and tables. DL, FY, and YR wrote the paper. YoZ, XL, and YiZ revised the manuscript. All authors have reviewed and approved the manuscript.

### Conflict of interest statement

The authors declare that the research was conducted in the absence of any commercial or financial relationships that could be construed as a potential conflict of interest.
